# Inversion of *ϕ*-OTDR Spatial Windowing Effects Using Wiener Deconvolution for Improved Acoustic Wavefield Reconstruction

**DOI:** 10.3390/s26051706

**Published:** 2026-03-08

**Authors:** Shangming Du, Tianwei Chen, Yuxing Duan, Ke Jiang, Song Wu, Can Guo, Lei Liang

**Affiliations:** 1 Sanya Science and Innovation Park, Wuhan University of Technology, Sanya 572000, China; shangming21@whut.edu.cn (S.D.); 307489@whut.edu.cn (Y.D.); 307396@whut.edu.cn (S.W.); guocan@whut.edu.cn (C.G.); 2 School of Safety Science and Emergency Management, Wuhan University of Technology, Wuhan 430062, China; chentianwei@whut.edu.cn; 3 National Engineering Research Center of Fiber Optic Sensing Technology and Networks, Wuhan University of Technology, Wuhan 430062, China; k.jiang@whut.edu.cn; 4 School of Mechanical and Electronic Engineering, Wuhan University of Technology, Wuhan 430062, China; 5 School of Computer Science and Artificial Intelligence, Wuhan University of Technology, Wuhan 430062, China

**Keywords:** distributed acoustic sensing, *ϕ*-OTDR, heterodyne detection, point spread function, Wiener filter, deconvolution, spatial spectrum estimation, SRP-PHAT

## Abstract

The spatial response of rectangular pulse heterodyne phase-sensitive optical time-domain reflectometry (ϕ-OTDR) to an acoustic event is characterized by a windowing function rather than a point-like sensitivity. This effect degrades the system’s spatial resolution and introduces systematic errors in array signal processing. This work presents modeling analysis and a mitigation strategy for this fundamental limitation. The spatial windowing effect is modeled as a point spread function (PSF) derived from physical mechanisms and system parameters, including the pulse width, gauge length, and intra-pulse intensity dynamics. The PSF model is validated against measurements under near-ideal conditions using a fiber-coupled tuning fork. A Wiener filter-based deconvolution method is utilized to invert the windowed spatial response towards a point-like response. The effectiveness of this inversion is demonstrated through enhanced spatial resolution and accurate reconstruction of two-dimensional wavefront geometry. Furthermore, the impact of this effect on array signal processing is quantitatively evaluated. The results demonstrate that the proposed method effectively suppresses systematic errors in wavefield analysis, and specifically enhances the accuracy and confidence of steered response power—phase transform (SRP-PHAT) spatial spectrum estimation. This study provides a systematic framework for understanding, quantifying, and inverting the spatial response in ϕ-OTDR, enabling accurate and interpretable acoustic field sensing.

## 1. Introduction

**Figure 1:**
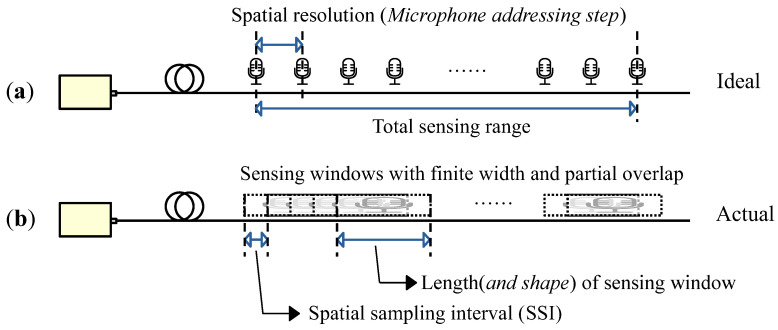
Spatial response characteristics of ϕ-OTDR. (**a**) Ideally independent point receivers; (**b**) actual sensitive windows with a certain spatial length, shape, and overlapping structure.

Distributed acoustic sensing (DAS) systems are conventionally abstracted as a continuous array of virtual microphones: the fiber optic cable is regarded as an array of independent point receivers along its length, each independently capturing acoustic signals, and the virtual microphones are synchronously recorded by the demodulation system (Figure 1a). Such high-level analogy has been proven effective in large-scale, long-range monitoring applications, including pipeline surveillance, railway security, and seismic acquisition.

However, this idealized point-receiver model becomes inadequate when addressing fine geometry of wavefronts, especially when the acoustic wavelength is comparable to or smaller than the spatial extent of the sensing channel [1,2]. In reality, each DAS channel acts as a spatially extended “sensitive window”, whose output represents a weighted coherent integral of acoustic pressure over its length and shape, as shown in Figure 1b. This spatial averaging—arising from the finite duration of the probe pulse and the demodulation process, introduces a blurring effect analogous to a spatial low-pass filter [3]. Consequently, wavefront geometry is obscured, time-delay estimations become biased, and the performance of advanced processing methods, such as vector acoustic analysis or high-resolution spatial spectrum estimation, is fundamentally compromised [4].

DAS data hold potential for two- or even three-dimensional acoustic field reconstruction if array data can be properly exploited [5,6,7,8]. Classical array processing algorithms, including delay-and-sum (DS) beamforming, minimum variance distortionless response (MVDR) beamforming, steered response power—phase transform (SRP-PHAT) beamforming [7,9]—and Multiple Signal Classification (MUSIC) [5,10], inherently assume ideal point-like sensors. Direct application of these methods to raw DAS measurements therefore leads to systematic errors and degraded localization accuracy. Spatial acoustic field sensing performance could be improved if the windowing effects are properly compensated.

Interestingly, The spatial blurring in DAS shares strong mathematical similarities with the convolution degradation problems (defocusing, motion blur, etc.) in optical imaging, where the system’s spatial response can be described by a point spread function (PSF) [11]. In image processing, deconvolution techniques such as Wiener filtering [12] and the Richardson–Lucy algorithm [13] have been widely adopted to recover obscured details and enhance resolution. Also, there has been research on using image deconvolution processing methods to improve acoustic beamforming [14,15]. This correspondence provides a valuable conceptual and methodological foundation for addressing spatial blurring in DAS.

In this work, we investigate the spatial blurring characteristics of a heterodyne, rectangular-pulse DAS system, deriving and experimentally validating its effective spatial response. We further examine how this blurring influences acoustic measurements and evaluate the potential of deconvolution-based restoration to mitigate these effects, thereby enhancing the fidelity of wavefield reconstruction and source localization capabilities.

## 2. Physical Mechanism and System Model

### 2.1. Causes of Degradation

The ϕ-OTDR system consists of three cascaded linear operations: (1) the external strain ϵ(z) is accumulated through the photoelastic effect and transformed into the phase ϕ(z) detected by the system, which is equivalent to an integral operation; (2) the phase signal is spatially averaged using a finite pulse width; and (3) the local derivative (difference) of the instantaneous (absolute) phase signal is computed to localize the disturbance.

#### 2.1.1. Width of the Probe Pulse

The ϕ-OTDR system employs a probe pulse with a finite width τ, corresponding to a spatial length within the fiber given by:

(1) $$L_{p}=\frac{c\cdot \tau }{2n}$$

where *c* is the speed of light in vacuum, and *n* is the refractive index of the fiber. The factor of 2 accounts for the round-trip propagation of light within the fiber.

An infinitely short pulse would generate a Rayleigh backscattering (RBS) signal from an exact spatial point, while a finite-width pulse can be considered as the superposition of an infinite number of such infinitesimally short pulses. At a given time *t*, the received signal is contributed by all points within the interval $\left(z_{1},z_{2}\right)$ where

(2) $$z_{1}=\frac{c\cdot (t-\tau /2)}{2n},\;z_{2}=\frac{c\cdot (t+\tau /2)}{2n}$$

along the fiber, resulting from the coherent superposition of RBS signals.

Therefore, considering only the finite pulse width, the system’s response to a point disturbance is represented by a normalized rectangular function:

(3) $$h_{p}\left(z^{\prime }\right)=\left\{\begin{array}{cc} \frac{1}{L_{p}} & \mathrm{when}\;-\frac{L_{p}}{2}\leq z^{\prime }\leq \frac{L_{p}}{2},\\ 0 & \mathrm{otherwise}. \end{array}\right.$$

The physical interpretation of this function is that a scattering particle only contributes to the received signal when illuminated by the probing pulse.

#### 2.1.2. Width of the Difference Operator (Gauge Length)

In ϕ-OTDR, when a disturbance occurs at a given location, the induced strain modulates the optical phase of not only that position but also all downstream segments of the sensing fiber. Consequently, the demodulated phase at a given point represents the cumulative integral of all applied strains from the fiber origin to that location. This type of sensing characteristic is generally not desired in practice, since the objective of distributed acoustic sensing is to localize disturbances such that the retrieved signal at a target position reflects only the acoustic field at that location. A differential operator, often mentioned as “gauge”, is utilized to recover the local strain by computing the spatial derivative of the accumulated phase response. This operation causes the response to a point disturbance to distribute as an antisymmetric function which resembles a first-order differential operator:

(4) $$h_{g}\left(z^{\prime }\right)=\frac{1}{L_{g}}\cdot \left[\delta \left(z^{\prime }+\frac{L_{g}}{2}\right)-\delta \left(z^{\prime }-\frac{L_{g}}{2}\right)\right]$$

where δ(·) is the Dirac function.

According to the homodyne coherence requirement in interferometric measurements, coherent interference is ensured only for Rayleigh backscattered fields originating from the same pulse and lying within the laser coherence length. The gauge length must not be smaller than the effective pulse length; otherwise, the differential operation would involve the superposition of partially overlapping scattering fields originating from different excitation intervals, and the resulting phase difference no longer possesses a well-defined physical meaning and instead manifests as random and unpredictable phase noise. Also, the gauge length also serves as a means of spatial averaging that regularizes the differential operation, preventing the differentiation kernel from uncontrollably amplifying the noise.

#### 2.1.3. Intra-Pulse Intensity Dynamics

In ϕ-OTDR, the amplified optical pulse exhibits a distinctive dynamic characteristic where the leading edge is brighter and the trailing edge is darker. This intra-pulse intensity non-uniformity arises from the transient gain dynamics of the erbium-doped fiber amplifier (EDFA) under high-power pulsed operation.

When the short-duration pulse (on the order of 100 ns) enters the EDFA, it rapidly induces stimulated emission from the inverted Er^3+^ ions at the ^4^$I_{13/2}$ metastable level. Since the pulse width is much shorter than the ion lifetime at this level (around 10 ms) and the pump cannot replenish the inverted population sufficiently during the pulse, the population inversion $N_{2}$ is significantly depleted during the pulse. According to the relationship:

(5) $$G\propto N_{2}$$

The gain of the EDFA decreases dynamically within the pulse duration, resulting in higher amplification at the leading edge and progressively reduced amplification toward the trailing edge.

**Figure 2:**
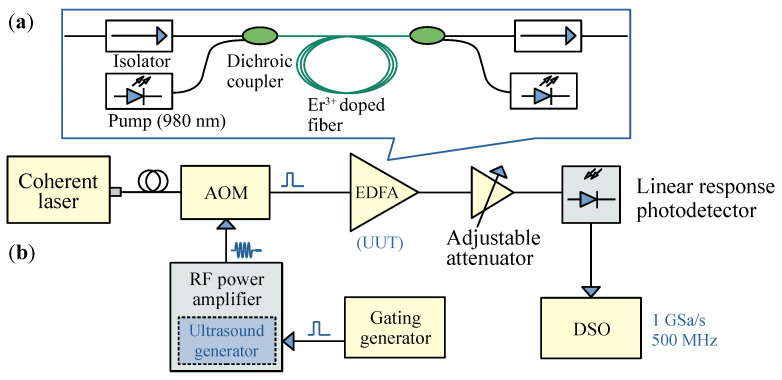
Experimental setup for transient dynamic testing of EDFA. (**a**) Internal optical path of bidirectional pumped EDFA; (**b**) optical path for dynamic characteristic testing. AOM: acousto-optic modulator; EDFA: erbium-doped fiber amplifier; UUT: unit under test; DSO: digital storage oscilloscope.

**Figure 3:**
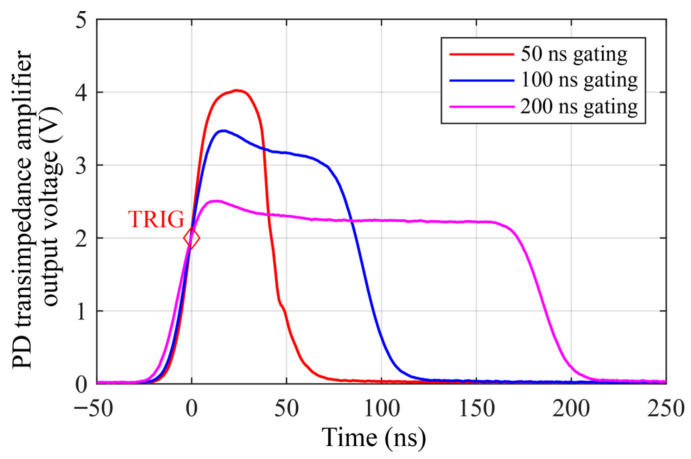
EDFA dynamic response under different pulse width conditions, averaged from 64 successive measurements.

The experimental setup was designed as shown in Figure 2. The probe pulse output from the modulator is amplified by the EDFA under test, and the power is adjusted to the linear range of the photodetector. A gating generation circuit sends a periodic pulse signal with a pulse width of 50–200 ns and a repetition rate of 20 kHz to the RF power amplifier with an integrated ultrasound generation circuit. The output electrical signal of the photodetector is recorded using an oscilloscope with 500 MHz analog bandwidth and 1 GSa/s sampling rate. The function of EDFA power with respect to time under different pulse width conditions is shown in Figure 3.

It can be observed from Figure 3 that the EDFA response power exhibits a characteristic of a bright leading edge and a dark trailing edge, consistent with theoretical expectations. Also: (1) After the application and removal of the drive signal, the emission intensity gradually increases and then decreases, eventually reaching a steady state or fully extinguishing after a finite time. This indicates that the system cannot respond instantaneously to external excitation. The actual emission duration is slightly longer than the gate time. (2) For gate signals of different durations, the steady-state response power differs, with longer gate times resulting in lower steady-state power. The energy level of the pulsed pumping affects the population inversion $N_{2}$, thereby influencing the gain of the EDFA.

The response sequence shown in Figure 3 represents the actual optical pulse power envelope injected into the sensing fiber; after conversion, it can represent the instantaneous power distribution within the spatial region occupied by the internal optical pulse of the sensing fiber at a certain moment. In principle, the Rayleigh-scattered electric field $E_{\mathrm{RS}}\left(t\right)$ received by the balanced photodetector from the ϕ-OTDR optical path is a coherent superposition of the electric fields contributed by all illuminated scatterers (assuming *N* scatterers):

(6) $$E_{\mathrm{RS}}\left(t\right)\propto \sum_{i=1}^{N}a_{i}\cdot \exp\left(j\cdot \phi_{i}\right)$$

The demodulated output phase angle is:

(7) $$\phi \left(z\right)=\arg(E_{\mathrm{RS}}\left(z\right))$$

where $a_{i}$ represents the contribution of the scatterers to the measured instantaneous phase, weighted according to the square root of the optical power(electric field amplitude) and the scattering intensity $\gamma_{i}$:

(8) $$a_{i}\propto \sqrt{\gamma_{i}\cdot P\left(z_{i},t\right)}$$

where $\gamma_{i}$ is the scattering intensity coefficient of the *i*-th scatterer, $P(z_{i},t)$ is the instantaneous power of the light pulse at position $z_{i}$ at time *t*, and $\phi_{i}$ is the intrinsic phase generated by the *i*-th scatterer, affected by local strain.

Rayleigh scattering in single-mode fiber is caused by microscopic refractive index inhomogeneities. These inhomogeneities are randomly distributed in space, causing $\sqrt{\gamma_{i}}$ to microscopically follow a Rayleigh distribution: that is, most $\gamma_{i}$ fluctuate around the average value, with occasional very strong or very weak reflection points. Statistically and macroscopically, single-mode fiber is considered a homogeneous medium, meaning that the statistical expectation $E\left[\gamma_{i}\right]$ of $\gamma_{i}$ is a constant in space. When examining the effect of pulse shape, the average weight can be considered as:

(9) $$w_{i}\propto E\left[\sqrt{\gamma_{i}}\cdot \sqrt{P(z_{i})}\right]=E\left[\sqrt{\gamma_{i}}\right]\cdot \sqrt{P(z_{i})}$$

Since $E\left[\gamma_{i}\right]$ is a constant, acting as a global scaling factor, it does not affect the relative weight distribution. The shape of the relative weight is entirely determined by $P(z_{i})$; therefore,

(10) $$W\left(z\right)=\sqrt{P(z)}$$

can be safely used as a weighting function for the contribution of the *z* position to the instantaneous phase of the demodulated output.

**Figure 4:**
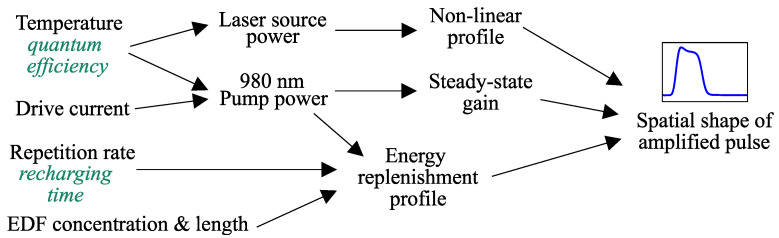
The combined influence of multiple factors on the spatial shape of a pulse.

The shape of the amplified pulse is governed by the interplay between input pulse energy, pump power, recovery time, and amplifier parameters such as erbium concentration and effective length, etc. Figure 4 illustrates some potential influence factors.

The abovementioned EDFA response characteristic is used in the derivation of a point spread function model in the next section. Recalibration of the model may be required whenever these parameters change appreciably.

### 2.2. The Point Spread Function Model

In signal processing, the characteristics of a linear time-invariant (LTI) system can be described by its impulse response. Extending this to linear space-invariant systems (sensor arrays), the system’s response to an ideal geometric point in the spatial domain is called the point spread function (PSF). PSF is frequently used in microscopic imaging. In optical systems, light emitted from an ideal point source, after passing through the imaging system, cannot be perfectly focused into a point again due to various inherent imperfections; this ideal point appears as a blurred spot in the final image. The one-dimensional or two-dimensional brightness distribution function of this spot is the PSF of the imaging system. A narrow and sharp PSF means that the optical system can resolve finer details. Similar to the characteristics of LTI systems, the image output by an imaging system is the convolution of the original object and the PSF. By measuring, estimating, or deriving the PSF of an imaging system, the blurring process can be reversed to some extent using deconvolution, recovering a clearer image closer to the original object from the blurred original sensor image.

Assuming that the sensing fiber or cable in the object under study has macroscopically uniform phase modulation characteristics, the DAS system can be regarded as a linear space-invariant system. The PSF is used to quantitatively model the blurring effect during the DAS’s observation of acoustic waves. The goal is to find a function $h_{\mathrm{RP}}\left(z^{\prime }\right)$ such that the signal observed by the DAS can be represented as a convolution of the perturbation signal $h_{\mathrm{RP}}\left(z^{\prime }\right)$:

(11) $$m\left(z\right)=s\left(z\right)*h_{\mathrm{RP}}\left(z\right)=\int_{-\infty }^{\infty }s\left(z^{\prime }\right)\cdot h_{\mathrm{RP}}\left(z-z^{\prime }\right)dz^{\prime }$$

#### 2.2.1. Considering Ideal Rectangular Pulse

With ϕ-OTDR, the accumulated phase caused by a point strain δ(z) is given by:

(12) $$\phi \left(z\right)=\frac{4\pi n\xi }{\lambda }\int_{0}^{z}\delta \left(\zeta \right)d\zeta =\frac{4\pi n\xi }{\lambda }\cdot H\left(z\right)$$

where H(z) is the Heaviside step function. This indicates that the system response from strain to phase is an integrator, and the effect of an ideal point strain on the lightwave phase is a step function. The entire measurement process can be viewed as first performing spatial averaging through the optical pulse and then applying spatial differentiation via the baseline processing. In a linear system, the total response of two independent operations is the convolution of their individual impulse responses:

(13) $$\begin{array}{cc} h_{\mathrm{RP}}\left(z^{\prime }\right) & =\left[\int_{-\infty }^{z}h_{p}\left(\zeta \right)d\zeta \right]*h_{g}\left(z\right) \end{array}$$

(14) $$\begin{array}{cc} \; & =\int_{-\infty }^{z}\left[h_{p}\left(\zeta \right)*h_{g}\left(\zeta \right)\right]d\zeta \end{array}$$

where $h_{p}\left(\cdot \right)$ and $h_{g}\left(\cdot \right)$ represent the contributions of the pulse width and gauge length to the blur kernel respectively. Substituting (3) and (4), we obtain:

(15) $$h_{\sigma }\left(z^{\prime }\right)=\frac{1}{L_{g}}\cdot \left[h_{p}\left(z^{\prime }+\frac{L_{g}}{2}\right)-h_{p}\left(z^{\prime }-\frac{L_{g}}{2}\right)\right]$$

Under the condition that the pulse shape is an ideal rectangle, the combined PSF of the sensing system is:

(16) $$h_{\mathrm{RP}}\left(z\right)=\int_{-\infty }^{z}h_{\sigma }\left(\zeta \right)d\zeta =\frac{1}{L_{g}}\int_{z-\frac{L_{g}}{2}}^{z+\frac{L_{g}}{2}}h_{p}\left(\zeta \right)d\zeta$$

This integral can be expressed in analytical form as a piecewise function:

(17) $$h_{\mathrm{RP}}\left(z\right)=\left\{\begin{array}{cc} 0 & \mathrm{when}\;z^{\prime }<-\frac{L_{g}+L_{p}}{2}\\ \frac{1}{L_{g}L_{p}}\left(z^{\prime }+\frac{L_{g}+L_{p}}{2}\right) & \mathrm{when}\;-\frac{L_{g}+L_{p}}{2}\leq z^{\prime }<-\frac{L_{g}-L_{p}}{2}\\ \frac{1}{L_{g}} & \mathrm{when}\;-\frac{L_{g}-L_{p}}{2}\leq z^{\prime }<\frac{L_{g}-L_{p}}{2}\\ -\frac{1}{L_{g}L_{p}}\left(z^{\prime }-\frac{L_{g}+L_{p}}{2}\right) & \mathrm{when}\;\frac{L_{g}-L_{p}}{2}\leq z^{\prime }<\frac{L_{g}+L_{p}}{2}\\ 0 & \mathrm{when}\;z^{\prime }\geq \frac{L_{g}+L_{p}}{2} \end{array}\right.$$

It is evident that this function represents a trapezoidal shape when $L_{g}>L_{p}$, or a triangular shape when $L_{g}\approx L_{p}$. This model quantitatively describes the response of the DAS system to any spatial disturbance field s(z). The observed signal m(z) is the result of convolving the true disturbance field s(z) with the point spread function $h_{\mathrm{RP}}\left(z\right)$.

#### 2.2.2. Considering EDFA Dynamics

Based on the measurements of the transient operating characteristics of the EDFA in Section 2.1.3, the optical pulse injected into the sensing fiber does not have a uniform brightness distribution in the time domain. Considering this, the rectangular optical pulse model described in Equation (3) needs to be extended to a more general arbitrary shape $h_{E}\left(z^{\prime }\right)$, with the total pulse energy normalized:

(18) $$\int_{-\infty }^{\infty }h_{E}\left(z^{\prime }\right)dz=1$$

and the support set remains symmetric about $z^{\prime }=0$:

(19) $$h_{E}\left(z^{\prime }\right)\neq 0\;\mathrm{if}\;\mathrm{and}\;\mathrm{only}\;\mathrm{if}\;z^{\prime }\in \left[-\frac{L_{p}}{2},\frac{L_{p}}{2}\right]$$

The derivation process of the point spread function is given in Appendix A of this article. It can be ultimately expressed as:

(20) $$\begin{array}{cc} h_{\mathrm{EP}}\left(z\right) & =\int_{-\infty }^{z}\left(h_{E}*h_{g}\right)\left(\zeta \right)\;d\zeta \end{array}$$

(21) $$\begin{array}{cc} \; & =\frac{1}{L_{g}}\left[H_{E}\left(z^{\prime }+\frac{L_{g}}{2}\right)-H_{E}\left(z^{\prime }-\frac{L_{g}}{2}\right)\right]. \end{array}$$

where

(22) $$H_{E}\left(z\right)=\int_{-\infty }^{z}h_{E}\left(\tau \right)\;d\tau .$$

Solving integral (20) as a piecewise function, we have:

(23) $$h_{\mathrm{EP}}\left(z\right)=\left\{\begin{array}{cc} 0 & \mathrm{if}\;z+\frac{L_{g}}{2}<-\frac{L_{p}}{2},\\ \frac{H_{E}\left(z+\frac{L_{g}}{2}\right)-H_{E}\left(z-\frac{L_{g}}{2}\right)}{L_{g}} & \mathrm{if}\;-\frac{L_{p}}{2}\leq z+\frac{L_{g}}{2}\leq \frac{L_{p}}{2},\\ 0 & \mathrm{if}\;z-\frac{L_{g}}{2}>\frac{L_{p}}{2}. \end{array}\right.$$

**Figure 5:**
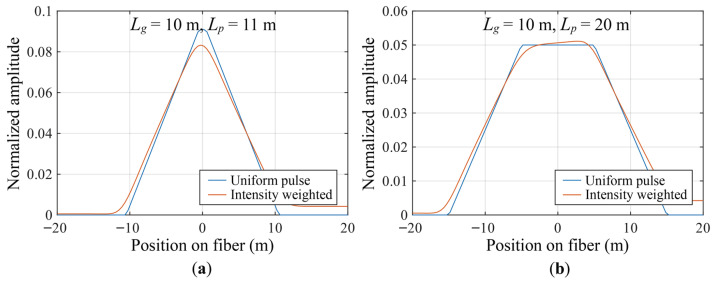
System point spread function of ϕ-OTDR. (**a**) Case where the differential window is slightly larger than the pulse width; (**b**) case where the differential window is twice the pulse width.

Under the condition of a pulse width $L_{p}=10\;m$, numerical solutions for the point spread functions $h_{\mathrm{RP}}\left(z\right)$ and $h_{\mathrm{EP}}\left(z\right)$ are calculated using common differential window selections, including cases where the difference window is slightly larger than the pulse width ($L_{g}=11\;m$) and where it is an integer multiple of the pulse width ($L_{g}=20\;m$). The spatial shapes of the resulting PSFs are shown in Figure 5.

Observing the final model, a sharp acoustic source is blurred into a triangular or trapezoidal shape with a width of approximately $L_{g}+L_{p}$. This model defines the ultimate spatial resolution of DAS, indicating that DAS has difficulty resolving two strain events separated by less than $(L_{p}+L_{g})/2$.

### 2.3. Validation of the Model

**Figure 6:**
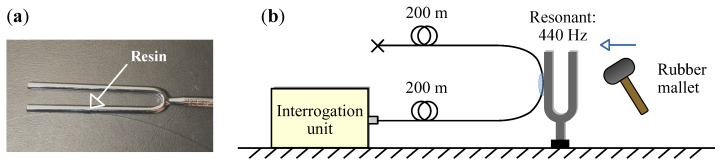
Design of an experimental setup for determining the point response function using the local mechanical excitation method. (**a**) The design drawing; (**b**) photograph of the coupled turning fork and optical fiber.

To verify the accuracy of the PSF derived above, the point response function was experimentally measured using the digital ϕ-OTDR interrogation setup reported in the authors’ previous work [16]. Since an ideal point strain source with infinitesimal spatial extent and sufficient energy is not attainable in the real-world, the experiment was designed to localize the disturbed region on the optical fiber as much as possible, and the diffusion characteristics of the point response were analyzed from the demodulated output (see Figure 6).

**Figure 7:**
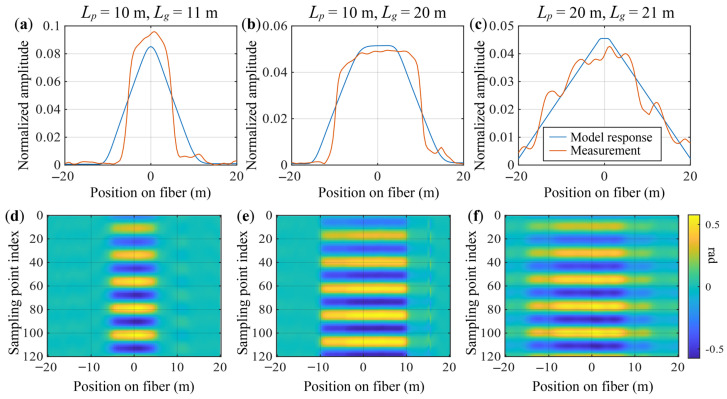
Array audio signals obtained from software demodulation of the tuning fork point perturbation RBS signal and their analysis results. (**a**–**c**) Amplitude feature extraction of the audio signals and comparison with the theoretical point spread function; (**d**–**f**) “Waterfall” plots of the differential-phase time series in the region of the disturbance point.

The pulse width was set to 96 ns (corresponding to a spatial width of 10 m) and 193 ns (corresponding to 20 m), respectively. A tuning fork was struck with a rubber mallet to initiate vibration. After waiting several seconds for the overtones to dissipate, data acquisition began from the output bus of the analog-to-digital converter (ADC). The gauge length was set to 11 m and 20 m for 10 m pulse width, and to 21 m for the 20 m pulse width. Demodulation operations (double conversion—zero intermediate frequency demodulation method, incremental DC-rejected phase unwrapper) were run respectively. The resulting acoustic signals are shown in Figure 7b.

To obtain an amplitude distribution curve of the audio signal analogous to the PSF, the following steps are performed for amplitude feature extraction from each audio segment:

1. A 150-order finite impulse response (FIR) band-pass filter is designed and applied to each DAS data channel to retain the characteristic frequency band at 440 Hz, yielding the filtered signal $x_{m}\left[n\right]$.
2. Reference sine and cosine signals are constructed:
  (24) $$r_{I}\left(t\right)=\cos\left(2\pi f_{0}t\right),\;r_{Q}\left(t\right)=\sin\left(2\pi f_{0}t\right)$$
  The inner product of each DAS channel signal with the reference signals is computed:
  (25) $$I_{m}=\frac{2}{N}\sum_{n=0}^{N-1}x_{m}\left[n\right]\;r_{I}\left[n\right],\;Q_{m}=\frac{2}{N}\sum_{n=0}^{N-1}x_{m}\left[n\right]\;r_{Q}\left[n\right]$$
3. The signal envelope amplitude for the *m*-th channel is calculated and stored:
  (26) $$A_{m}=\sqrt{I_{m}^{2}+Q_{m}^{2}}$$
4. Both the PSF model and the measured amplitude curve $A_{m}$ are normalized using trapezoidal numerical integration so that the area under each curve is equal, thereby removing the influence of absolute signal energy on shape comparison.

Figure 7a presents a comparison between the normalized audio signal amplitude distribution (measured response) and the theoretical PSF (model response). Two metrics are employed to quantitatively assess their similarity: Pearson correlation coefficient, which evaluates the overall shape resemblance and the full width at half maximum (FWHM), which provides a single-parameter measurement of the window width and directly determines the spatial resolution of the DAS system for localized events.

Table 1 presents the analysis results. Combined with Figure 7 it can be observed that, in terms of Pearson correlation coefficient and FWHM, the measured data and the theoretical model exhibit similar distribution shapes and width characteristics. Further observation reveals that the amplitude distribution on the positive half of the position axis of the window is higher than that on the negative half, which may confirm the influence of the EDFA dynamic process. In Figure 7a,b, the measured response does not completely coincide with the theoretical model, and Figure 7c even shows patterns of grating lobes. The main reason for this is the non-ideal nature of the point perturbation simulated by the mechanical excitation method, and the interference effect during the propagation of the mechanical wave along the optical fiber. This phenomenon also indicates that under real-world measurement conditions, DAS may exhibit more complex spatial window characteristics than the theoretical derivation in this section.

## 3. Wiener Deconvolution Algorithm

### 3.1. Reconstruction of Spatial Response Using Wiener Filter

According to the derivation in Section 2.2, the system response of DAS can be written as a spatial convolution:

(27) m(z)=s(z)∗h(z)+n(z)

where m(z) is the acoustic field directly observed by DAS, h(z) is the PSF, and n(z) is additive measurement noise.

If a filter g(z) is designed such that its output $\hat{s}\left(z\right)$ is the minimum-mean-square-error estimation of s(z), i.e.,

(28) $$\mathrm{minimize}:\;E\{|s\left(z\right)-\hat{s}\left(z\right){|}^{2}\}$$

the filter can then reverse a spatial window of DAS into a proximity point response.

The spatial convolution is equivalent to multiplication in the frequency domain. Transforming Equation (27) into the frequency domain yields:

(29) M(f)=S(f)·H(f)+N(f)

where *f* denotes spatial frequency.

The problem is thus reduced to designing a transfer function G(f) that estimates the point-response acoustic field $\hat{s}\left(f\right)$ from the observed signal M(f), i.e., solving for the optimal G(f) in the equation

(30) $$\hat{S}\left(f\right)=M\left(f\right)\cdot G\left(f\right).$$

If the inverse of the convolution is used directly as G(f):

(31) $$G\left(f\right)=\frac{1}{H(f)},$$

the problem becomes ill-posed because M(f) contains the noise term N(f). The inverse operation would significantly amplify noise, especially at or near frequencies where H(f) approaches zero, causing G(f) to tend toward infinity. Any slight measurement noise would be infinitely amplified, completely masking the target signal by noise.

Wiener deconvolution provides a linear method that achieves an optimal trade-off between deblurring and noise suppression. The idea is to introduce an estimation of noise-to-signal ratio (NSR) to stabilize the solution of deconvolution [12]. Its transfer function is defined as

(32) $$G\left(f\right)=\frac{1}{H(f)}\cdot \frac{{\left|H\left(f\right)\right|}^{2}}{{\left|H\left(f\right)\right|}^{2}+\frac{P_{n}\left(f\right)}{P_{s}\left(f\right)}},$$

where $P_{n}\left(f\right)=E\left\{{\left|N\left(f\right)\right|}^{2}\right\}$ is the average noise power spectrum, which can be measured from quiet observation signals; $P_{s}\left(f\right)=E\left\{{\left|S\left(f\right)\right|}^{2}\right\}$ is the average power spectrum of the original signal; and $\frac{P_{n}\left(f\right)}{P_{s}\left(f\right)}$ is the reciprocal of frequency-domain signal-to-noise ratio (SNR), denoted as noise-to-signal ratio (NSR). In frequency bands with high SNR,

(33) $${\left|H\left(f\right)\right|}^{2}\gg \frac{P_{n}\left(f\right)}{P_{s}\left(f\right)},$$

so that

(34) $$G\left(f\right)\approx \frac{1}{H(f)}\cdot \frac{{\left|H\left(f\right)\right|}^{2}}{{\left|H\left(f\right)\right|}^{2}}=\frac{1}{H(f)}.$$

Here the filter primarily performs deconvolution, recovering as much signal information as possible. In frequency bands with low SNR,

(35) $${\left|H\left(f\right)\right|}^{2}\ll \frac{P_{n}\left(f\right)}{P_{s}\left(f\right)},$$

leading to

(36) $$G\left(f\right)\approx \frac{1}{H(f)}\cdot \frac{{\left|H\left(f\right)\right|}^{2}}{\frac{P_{n}\left(f\right)}{P_{s}\left(f\right)}}\propto H\left(f\right).$$

In this case the filter gain is substantially reduced to suppressing noise in that band.

Because s(z) is unknown and may vary along the fiber, $P_{s}\left(f\right)$ is difficult to estimate. In practice, a parametric approach is often adopted, approximating the NSR as an adjustable parameter:

(37) $$G\left(f\right)\approx \frac{1}{H(f)}\cdot \frac{{\left|H\left(f\right)\right|}^{2}}{{\left|H\left(f\right)\right|}^{2}+\mathrm{NSR}}.$$

Choosing a smaller NSR makes the filter more aggressive, enhancing the deconvolution effect and recovering more details of the original signal at the cost of higher noise; choosing a larger NSR makes the filter more conservative, sacrificing detail recovery for lower-noise output.

### 3.2. Estimation of Hardware Resource Utilization

The role of Wiener deconvolution is to restore spatial blurring and correct phase errors in ϕ-OTDR measurements (discussed in the next section). The design objective of a DAS system is to output acoustic signals in real time; Wiener deconvolution must be implementable in hardware with real-time processing capability to be effective in practical engineering applications. Building upon the authors’ prior work on hardware-efficient ϕ-OTDR phase demodulation [16], an FPGA-based 16-bit processing datapath has been investigated to evaluate its resource overhead and integration feasibility (chip model XCZU47DR).

The FPGA-based Wiener deconvolution module consists of an FFT core, a frequency domain Wiener filtering unit, and an IFFT core. The Wiener filter coefficients $\frac{{\left|H\left(f\right)\right|}^{2}}{{\left|H\left(f\right)\right|}^{2}+\mathrm{NSR}}$ can be precomputed offline and stored in ROM, and only complex multiplications are performed online. FFT and IFFT have been maturely implemented and packaged as LogiCORE IP, and a maximum 4096-point butterfly operation can be achieved efficiently. To suppress temporal aliasing, the length of the linear convolution must not exceed the FFT size. With 250 MSa/s ADC, the spatial sampling interval (SSI) on the fiber is around 0.4 m. In this condition, 30 m of PSF kernel has 75 samples, and the datapath can process 4022 spatial samples in one-shot, which corresponds to around 1.6 km. For complex multiplication, each operation is essentially

(38) (a+jb)·(c+jd)=(a·c−b·d)+j(a·d+b·c)

which can be processed using 3 Digital Signal Processor 48-bit Multiply-and-accumulate (DSP48 MAC) cores. Based on this analysis, a hardware resource estimate can be provided (Table 2).

According to the delay estimation output of the design suite, with a pipelined, streaming I/O implementation, FFT and IFFT operations each require 8341 clock cycles. Complex multiplication can be parallelized with 8×3 DSP48 instances, thus requiring 512 clock cycles. In a typical 350 MHz clock domain, the entire algorithm has 49.125 µs pipeline delay, allowing at least a 20,000 pulses/s ϕ-OTDR measurement stream to be processed in real time. The above resources are used to process data from approximately 1.6 km of sensing fiber. If the fiber exceeds this length, multiple such datapaths may be instantiated to ensure real-time processing.

## 4. Impact and Inversion of Windowing Effects

This section utilizes numerical simulation experiments to evaluate negative impacts of spatial windowing on DAS measurements and to quantitatively evaluate the improvement through Wiener deconvolution.

### 4.1. One-Dimensional Measurement: Spatial Aliasing and Its Restoration

When considering situations where the disturbance source is located on an optical fiber or at a very close distance, the output signals of adjacent strain sensors alias, causing the nearest distance between the two resolvable strain events to be much larger than the spatial sampling interval (SSI).

The common form of the Wiener filter (Equation (37)) contains only a single-parameter NSR, used to control the filter’s aggressiveness and balance blur recovery effectiveness with noise amplification. Although its value does not require complex parameter estimation and adjustment, a correct estimation of NSR can achieve a useful balance. A simulation experiment is designed to explore the effects of different NSR selections on processed strain outputs.

**Figure 8:**
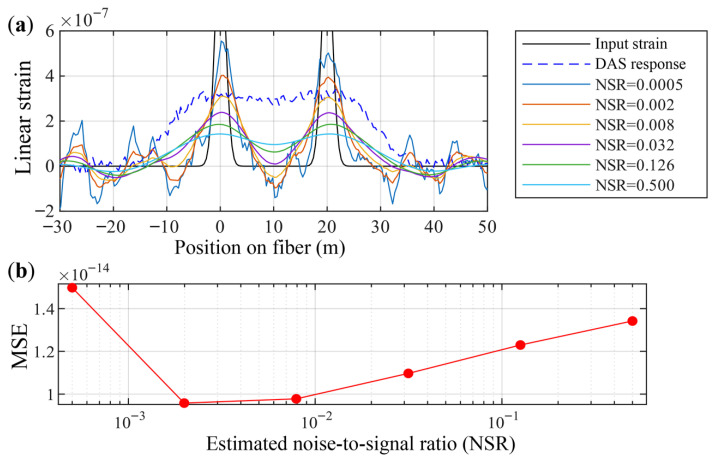
Strain fields under typical parameter conditions: the original strain field, the DAS-response strain field, and the output strain fields obtained using Wiener deconvolution with different NSR parameters. (**a**) Input and output strain fields for different NSR values; (**b**) relationship between the NSR parameter and the mean-square error.

Figure 8 shows, under typical DAS parameters ($L_{p}=10$, $L_{g}=20$), the DAS response obtained by numerically solving the point-spread convolution model for two narrow strain events at $z_{1}=0$ m and $z_{2}=20$ m, together with the output strain fields resulting from Wiener deconvolution with different NSR parameters.

Observing Figure 8a, when the NSR is set to very small values ($5\times 10^{-4}$, $2\times 10^{-3}$), the Wiener filter treats the input signal as a blurred but nearly noise-free signal. In this regime the filter acts aggressively, and the shape of the output strain field closely resembles the original strain field—i.e., the DAS output that originally appeared as a single trapezoidal-shaped region is resolved into two distinct strain events. However, an overly aggressive Wiener filter also significantly amplifies noise. When the NSR is chosen moderately (around $1\times 10^{-2}$), the valley between the two strain events approaches zero, the events remain clearly separated, and noise suppression is simultaneously achieved. Yet the event widths are somewhat broadened, indicating that, in the presence of noise, the recovered peaks still exhibit positional uncertainty compared with the sharp input strain. If the NSR is set large (0.5), the Wiener filter behaves conservatively. The deconvolved output then exhibits low noise, but the two strain events are not distinctly resolved.

Figure 8b shows that, in terms of the minimum mean-square error between the output signal and the true signal, there exists an NSR value that yields an optimal Wiener deconvolution.

**Figure 9:**
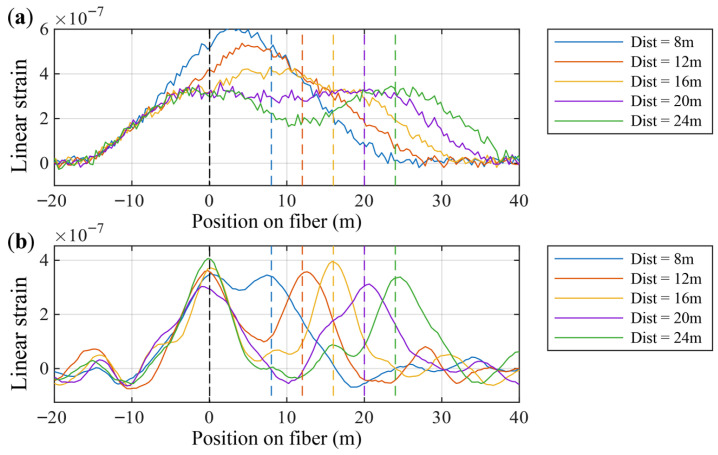
DAS system response to two closely spaced narrow strain events under the parameters $L_{p}=10$, $L_{g}=20$, and the corresponding Wiener deconvolution results. (**a**) Output of the convolution model (numerical solution with typical system noise); (**b**) deconvolution output (estimated NSR set to $5\times 10^{-3}$).

Figure 9a shows the noisy DAS measurement of two narrow strain events at different separations under typical parameters, computed by the convolution model with band-limited Gaussian noise added. The first event is fixed at $z_{1}=0$, while the second event is placed at $z_{2}\in \left\{8,12,16,20,24\right\}$; both events have the same amplitude. Figure 9b presents the results after Wiener deconvolution. Comparing the two figures reveals that the DAS measurement cannot resolve two strain events whose separation is smaller than $(L_{p}+L_{g})/2$. A discernible valley between the two strain peaks appears only when the event separation exceeds about 20 m. This occurs because although the system’s spatial distinguishability is examined for ideal Dirac events, the strain events used in simulation possess a finite width to ensure numerical stability.

Figure 9b shows Wiener deconvolution results, using a fixed $\mathrm{NSR}=5\times 10^{-3}$. When the two events are separated by 8 m, a valley between the two strain peaks can already be observed. For separations greater than 12 m, the two strain events become clearly resolvable.

**Figure 10:**
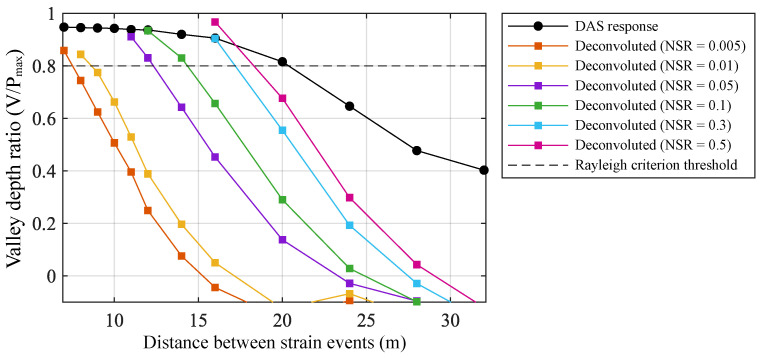
Average valley-depth ratio computed by the Monte-Carlo method as a function of NSR and event separation Δz under the parameters $L_{p}=10$, $L_{g}=20$.

In optics, the Rayleigh criterion is commonly employed as an empirical standard for judging whether two point-like optical targets are resolvable; this empirical criterion can be borrowed to quantitatively study the spatial resolution of DAS. Defining the valley-depth ratio of the output strain field and considering two events as resolvable when this ratio is less than 0.8, we take the event separation Δz and the NSR as independent variables. For each combination of Δz and NSR, 500 Monte-Carlo simulations are performed, and the average valley-depth ratio is plotted against Δz and NSR. The results are shown in Figure 10 and Table 3.

### 4.2. Two-Dimensional Measurement: Wavefront Phase Error and Its Restoration

The DAS cable constitutes a continuously distributed acoustic sensor array. Through beamforming methods, the observed signals can be processed to achieve 2D acoustic field measurement tasks such as spatial spectrum estimation. Such applications require the sensing elements to possess accurate phase response capability. Convolution degradation, however, exerts a more complex influence on the phase information of the acoustic field and must be specifically considered in signal modeling and processing.

In near-field or complex acoustic environments, the curvature of the acoustic wavefront cannot be neglected. An ideal point response should accurately reflect the local phase distribution of the wavefront. The convolution operation, however, performs a weighted average of acoustic signals from different spatial positions, causing the output acoustic phase at a given window to deviate from the theoretical point response at the center of that window. Especially when the distance from the sound source to the array is short, the DAS’s window function is not negligible compared to the acoustic wavelength. Under these conditions, the DAS observation signal will exhibit significant and complex phase errors, which degrade the accuracy of phase-based applications such as sound source localization.

The demodulated phase differential at position *z* is not the theoretical point response

(39) $$\Delta \phi_{\mathrm{norm}}^{\mathrm{ideal}}\left(z_{0},t\right)\propto p\left(z_{0},t\right),$$

but is approximately

(40) $$\Delta \phi_{\mathrm{norm}}\left(z_{0},t\right)\propto \int_{-\infty }^{\infty }p\left(z,t\right)\;h\left(z-z_{0}\right)\;dz,$$

where p(z,t) denotes the axial strain or equivalent acoustic pressure, and h(z) is the spatial window function determined by the pulse width and the differential window length. This indicates that the effect of DAS on the spatial acoustic field resembles a spatial low-pass and band-stop filter.

Suppose that in the far field region a sound wave impinges obliquely on the array. The acoustic field observed by the array is a projected wave propagating along the *z*-direction, whose axial spatial wavelength is

(41) $$\lambda_{z}=\frac{\lambda }{\cos\theta }.$$

The acoustic wave field on the array can be expressed as

(42) $$p\left(z,t\right)=A\cdot \cos\left(\omega t-k_{z}z+\phi_{0}\right),$$

where $k_{z}=k\cdot \cos\theta$ is the wavenumber projected onto the array along the *z*-direction. Substituting the plane wave into the convolution yields

(43) $$\Delta \phi_{\mathrm{norm}}\propto \int h\left(\xi \right)\cdot \cos\;\left(\omega t-k_{z}\cdot \left(z_{0}+\xi \right)\right)\;d\xi .$$

The integral can be written as

(44) $$\Delta \phi_{\mathrm{norm}}\left(z_{0},t\right)=\left|H(k_{z})\right|\cdot \cos\;\left(\omega t-k_{z}z_{0}+\arg\left(H\left(k_{z}\right)\right)\right).$$

This shows that the phase deviation of the acoustic wave in the DAS measurement output is not random noise, but a deterministic phase response of the spatial filter. When $\arg(H\left(k_{z}\right))=\pi$, the acoustic wave can undergo complete phase reversal.

First consider the simple case where the window function is rectangular with a width of $L_{w}$, i.e.,

(45) $$h_{\mathrm{rect}}\left(z\right)=\frac{1}{L_{w}}\cdot \mathrm{rect}\;\left(\frac{z}{L_{w}}\right).$$

Taking the Fourier transform of the rectangular function, the spatial-frequency response of DAS becomes

(46) $$H\left(k_{z}\right)=\mathrm{sinc}\;\left(\frac{k_{z}\cdot L_{w}}{2}\right).$$

Define $\eta =\frac{k_{z}\cdot L_{w}}{2}$. Its value determines the sign of the overall transfer function of DAS for a pure spatial sinusoidal mode. The condition for acoustic wave phase reversal is

(47) $$\eta =\frac{k_{z}\cdot L_{w}}{2}\in \left(\pi ,2\pi \right)\cup \left(3\pi ,4\pi \right)\cup \left(5\pi ,6\pi \right)\cup \ldots$$

**Figure 11:**
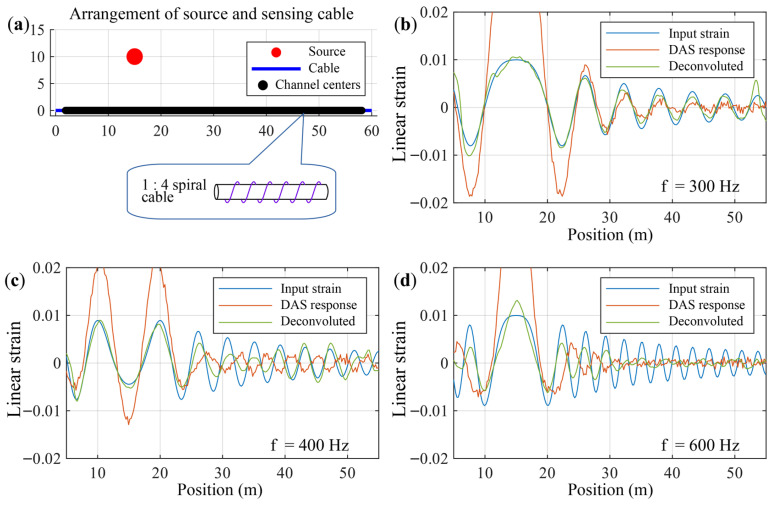
Arrangement of the sound source and DAS array, and the signal dynamics at the peak of the sound signal. (**a**) Geometric layout of the sound source and sensing cable, and structure of the spiral optical cable; (**b**–**d**) instantaneous strain, DAS response and deconvolved signal; (**b**) correct phase relationship at 300 Hz; (**c**) phase reversal at 400 Hz; (**d**) coherent cancellation at 600 Hz.

In the real-world, the ϕ-OTDR window function is not rectangular, and the sound source is often located in the near field, and the sound wave does not have a definite wavenumber on the optical cable. This leads to the possibility of simultaneous occurrences of correct phases, coherence cancellation, and phase reversal in the sensing data at a single moment. The following simulation demonstrates a single frequency steady state point sound source in the near field ($s=(x_{s},y_{s})$, $x_{s}=15$, $y_{s}=10$) and the axial strain it generates on a spiral optical cable. The layout of the sound source and DAS array is shown in Figure 11.

**Figure 12:**
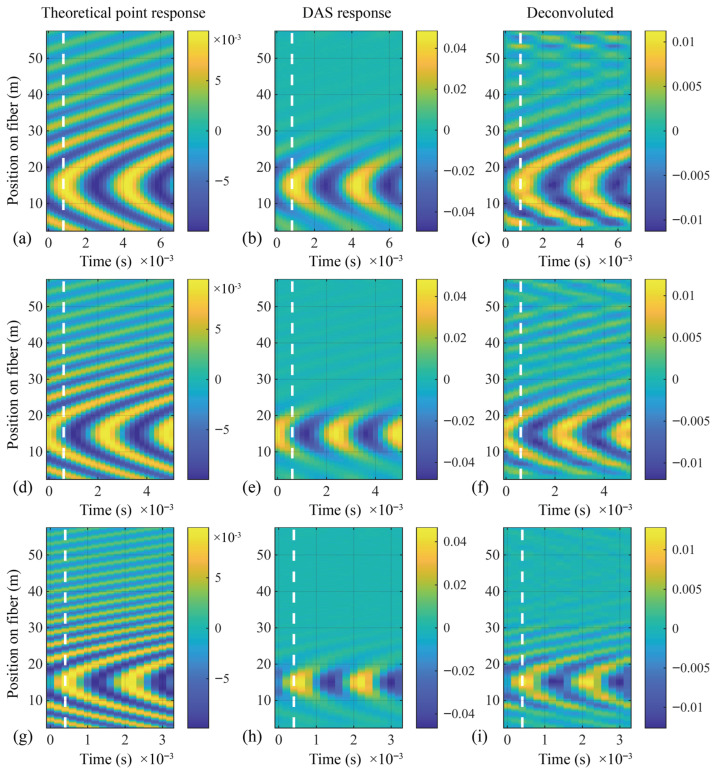
Two-dimensional data of axial strain generated on the sensing optical cable by a near-field single-frequency steady-state point sound source. (**a**–**c**): sound frequency 300 Hz; (**d**–**f**): sound frequency 400 Hz; (**g**–**i**): sound frequency 600 Hz; (**a**,**d**,**g**): theoretical point response as performance reference; (**b**,**e**,**h**): numerical solution of DAS output signal obtained by convolution model; (**c**,**f**,**i**): signal after Wiener deconvolution processing using NSR = 0.08.

Figure 12 presents the numerical results of the theoretical point response of the strain field, the DAS response, and the Wiener deconvolution output. The simulated source frequencies are chosen as 300 Hz, 400 Hz and 600 Hz to cover the cases of correct spatial phase, local coherent cancellation, and local phase reversal, respectively. The white dashed lines in the figure mark the time slice at which the acoustic signal reaches its peak; the instantaneous strain response and the deconvolution output at that moment are shown in Figure 11b–d. It can be observed that, in the presence of local coherent cancellation or phase reversal, the Wiener deconvolution operation (1) restores the correct amplitude of the signal at positions where acoustic waves undergo coherent superposition while suppressing measurement noise elsewhere (Figure 11b), and (2) recovers the reversed phases back to the correct phase (Figure 11c). However, when the signal suffers substantial coherent cancellation (Figure 11d and Figure 12i), the Wiener deconvolution shows limited recovery capability.

To quantitatively evaluate the recovery performance, the Pearson correlation coefficient is utilized as the metric. A correlation coefficient close to 1 indicates that two wave-field profiles (e.g., the spatial distribution of strain at a given time, or the time series at a given location) have similar shapes and trends. Hence this parameter can be used to assess the recovery of fine wave-front structures, i.e., the restoration of the wave’s phase and spatial distribution characteristics. DAS data possess both temporal and spatial dimensions; Pearson correlation coefficients can be computed separately along each dimension to provide different insights:

(1) Time dimension: For each time instant *t*, the correlation coefficient along the spatial dimension is calculated as

(48) $$R_{t}\left(t\right)=\mathrm{corrcoef}\left(\epsilon_{\mathrm{truth}}\left(\cdot ,t\right),\;\epsilon_{\mathrm{deconv}}\left(\cdot ,t\right)\right).$$

This index reflects how well the wavefront shape of the entire sensing array at a specific moment matches the true wavefront. By analyzing the variation of $R_{t}$ with time, the performance of the deconvolution can be observed at different stages of the wave, such as at peaks and zero-crossings.

(2) Spatial dimension: For each position *x* along the sensing cable, the correlation coefficient along the temporal dimension is calculated as

(49) $$R_{x}\left(x\right)=\mathrm{corrcoef}\left(\epsilon_{\mathrm{truth}}\left(x,\cdot \right),\;\epsilon_{\mathrm{deconv}}\left(x,\cdot \right)\right).$$

This index indicates how well the signal waveform received by the sensing element at a particular location matches the true waveform. By analyzing the variation of $R_{x}$ with spatial position, the recovery effectiveness of the deconvolution algorithm can be evaluated in different regions of the fiber, e.g., directly opposite, near, and far from the sound source.

**Figure 13:**
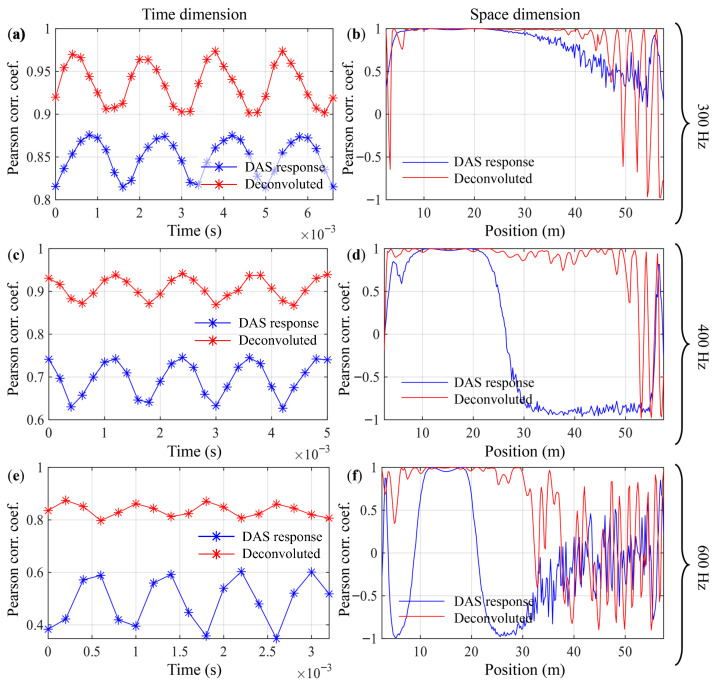
Pearson correlation coefficients of the original DAS output and the Wiener deconvolution output relative to the theoretical point response. (**a**,**b**): Source frequency 300 Hz; (**c**,**d**): 400 Hz; (**e**,**f**): 600 Hz. (**a**,**c**,**e**): Correlation coefficient computed along the spatial dimension for each time point; (**b**,**d**,**f**): correlation coefficient computed along the temporal dimension for each position on the sensing cable.

Figure 13 and Table 4 present the Pearson correlation coefficients in the temporal and spatial dimensions. Compared with the original DAS output, the signals processed by Wiener deconvolution exhibit higher Pearson correlation coefficients with the true strain field in both dimensions. From Figure 13a–f it can be observed that:

1. In the frequency band where coherent cancellation and phase reversal are not pronounced, the Pearson correlation coefficient of the DAS output decreases with increasing distance. This is mainly due to the attenuation of the acoustic wave during spatial propagation, which reduces the SNR. After Wiener deconvolution, the output signal shows overall improvement in the temporal dimension, indicating that the operation helps bring the actual window response closer to the theoretical point response, thereby enhancing the accuracy and linearity of the time series relative to the ideal point response. In Figure 13b, beyond a cable position of about 50 m, the Pearson correlation coefficient drops sharply. The reason is that as the SNR decreases, the chosen NSR becomes overestimated for that location, causing the Wiener filter to become unstable and exhibit noise amplification behavior.
2. Within the near field, sound waves are incident on the cable at an angle, and the local wavenumber increases with distance. In Figure 13d, at a position around 22 m, the correlation coefficient of the original DAS signal rapidly drops to zero and then rises to −1, which is a typical case of crossing the critical condition described by Equations (46) and (47). A Pearson correlation coefficient approaching −1 means that the DAS output signal is opposite to the theoretical point response. Under this critical condition, the deconvolved output consistently shows a correlation coefficient close to 1, demonstrating that Wiener deconvolution can recover the phase reversal caused by spatial aliasing of the window response.
3. In the high-frequency band, the wavenumber of the acoustic signal lies in a region where the spatial transfer function of the response window undergoes strong attenuation. In Figure 13f, the Wiener deconvolution output exhibits a larger usable spatial range compared with the original DAS output. At farther positions, e.g., around 40 m, both the original DAS signal and the deconvolved output show random and highly fluctuating correlation coefficients, indicating that the acoustic field cannot be reliably characterized by DAS under such conditions.

### 4.3. Improving Spatial Spectrum Estimation Using Wiener Deconvolution

Based on phased-array theory, DAS systems have the potential to reconstruct the spatial distribution of the acoustic field both along the fiber and in the transverse direction. Benefiting from dense spatial sampling and advanced spatial signal processing techniques, it is possible to enable target localization and tracking in two-dimensional or even three-dimensional space. This extends the one-dimensional linear sensing array into a system with angular resolution and spatial detection capabilities.

SRP-PHAT is a simple yet effective spatial spectrum estimation algorithm; it estimates a source location by scanning the space and summing phase-normalized inter-sensor correlations that are time aligned for each candidate position (further introduced in [9] and Appendix B). It also relies on the assumption of an ideal point response, which raw DAS data do not satisfy well. Numerical simulations are utilized to investigate the influence of the DAS window effect on the spatial spectrum and how it can be improved using Wiener deconvolution.

**Figure 14:**
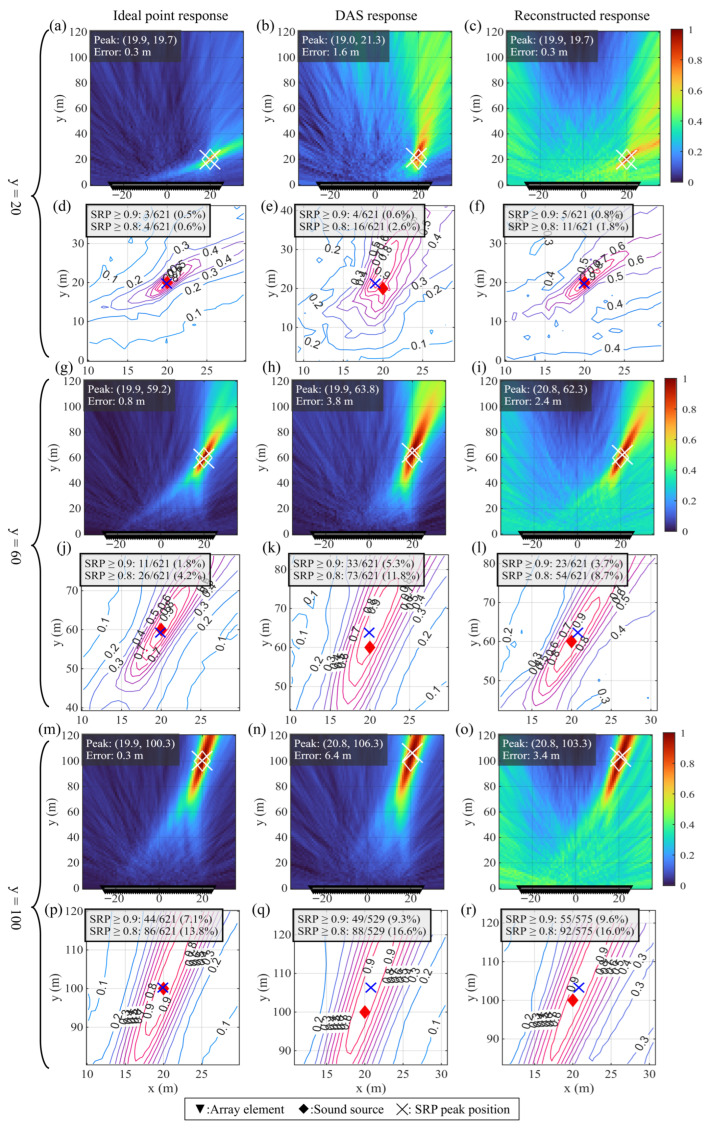
SRP spatial spectrum calculated under the condition that the narrowband sound source is located in the near field. (**a**–**c**,**g**–**i**,**m**–**o**): Wide-area scan results plotted using colored heatmap; (**d**–**f**,**j**–**l**,**p**–**r**): Contour curves of local area near the SRP peak; (**a**,**d**,**g**,**j**,**m**,**p**): Spatial spectrum computed using ideal point response; (**b**,**e**,**h**,**k**,**n**,**q**): Spatial spectrum computed using DAS response; (**c**,**f**,**i**,**l**,**o**,**r**): Spatial spectrum computed using reconstructed response; (**a**–**f**): Source distance to array is 20 m; (**g**–**l**): Source distance to array is 60 m; (**m**–**r**): Source distance to array is 100 m.

A simulation experiment was designed and conducted using the parameters in Table 5. The array aperture was chosen to be 50 m, the sound source position was set at x=20 m, and *y* was adjusted within a certain distance in the near field (Figure 14).

Results show that when the sound source is located in the very near field, the SRP spatial spectrum constructed by the ideal point array exhibits a sharp single peak and has high location confidence. However, the spatial spectrum constructed based on the DAS response shows significant main lobe broadening in the direction perpendicular to the array and introduces a high-confidence region on the order of 10 m, leading to an increase in the localization system error. After adopting the sound field reconstruction method based on Wiener deconvolution, the main lobe of the spatial spectrum is narrowed, and its shape is closer to the ideal point response. The absolute localization error is reduced from 1.6 m to 0.3 m, and the proportion of high-confidence (0.9, 0.8) regions is reduced, indicating that the spatial resolution is improved. As the distance of the sound source increases to a level comparable to or greater than the array aperture, the system error and defocusing effect caused by DAS are further aggravated. The reconstruction method can still reduce the localization error to some extent (from 3.8 m to 2.4 m, and from 6.4 m to 3.4 m), but the spatial spectrum gradually shows a ridge-like structure extending along the arrival direction, indicating that the two-dimensional position estimation gradually degenerates into the arrival direction estimation, and the improvement effect of sound field reconstruction weakens with increasing distance.

To further quantify the improvement of signal reconstruction on SRP-PHAT spatial spectrum estimation performance under DAS linear array conditions, a numerical simulation experiment based on the Monte-Carlo method was designed for statistical analysis of localization errors. Under fixed sound source and array geometry, observation data containing random noise were independently and repeatedly generated. Both the original DAS response and the reconstructed signal were processed, and descriptive statistics and distribution visualization of the accumulated localization error samples were performed. The experiment selected the parameters listed in Table 5, setting the vertical distance from the sound source to the array to 20 m, 40 m, 60 m, 90 m, and 120 m to fully cover the sensing distance from the very near field to the near field. After each iteration of the spatial spectrum scan, the peak position of the normalized SRP matrix and its Euclidean distance from the actual sound source position was calculated as the localization error. After 100 Monte-Carlo runs, the stored localization error vector was analyzed using statistical methods.

**Figure 15:**
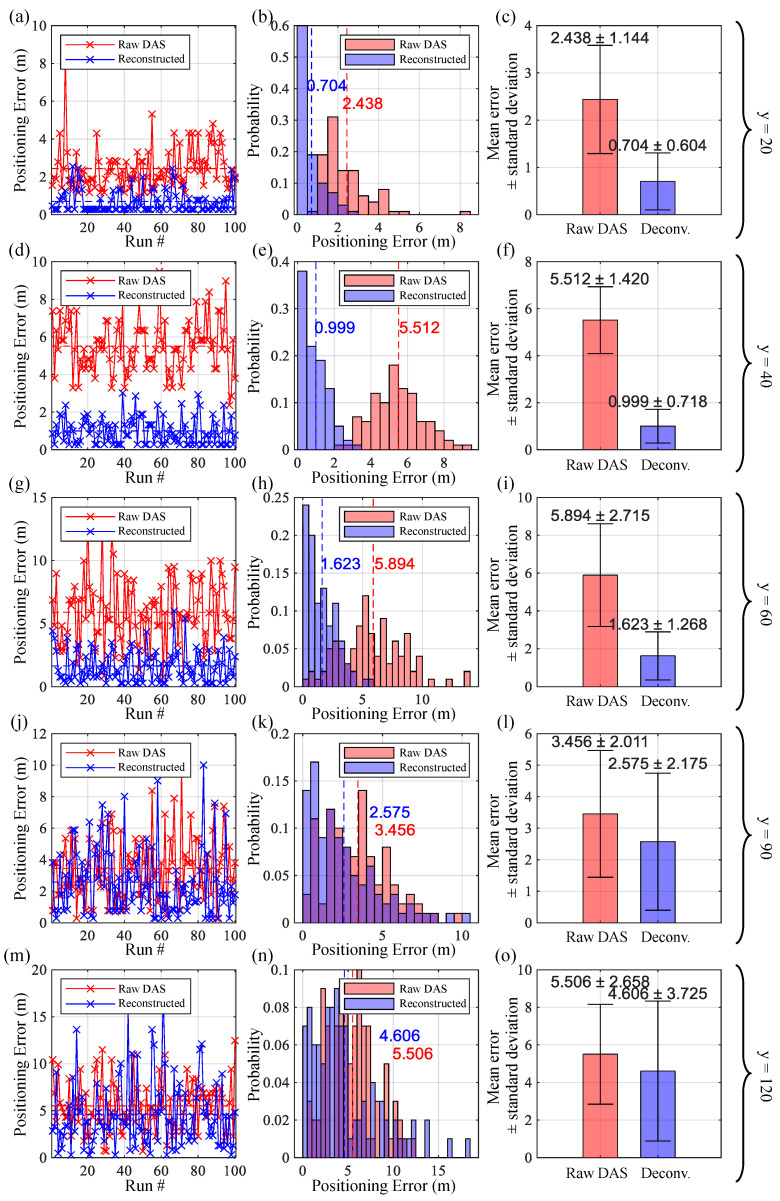
Results of Monte-Carlo experiments for single-source localization. (**a**,**d**,**g**,**j**,**m**): changes in positioning error before and after deconvolution processing with the number of Monte-Carlo runs; (**b**,**e**,**h**,**k**,**n**): distribution of errors; (**c**,**f**,**i**,**l**,**o**): mean and standard deviation of errors; (**a**–**c**): Source distance to array is 20 m; (**d**–**f**): Source distance to array is 40 m; (**g**–**i**): Source distance to array is 60 m; (**j**–**l**): Source distance to array is 90 m; (**m**–**o**): Source distance to array is 120 m.

Figure 15 presents the results in three ways: a line graph of the number of runs versus the positioning error, a histogram of the positioning error distribution, and a mean-variance graph. It can be observed that:

1. In the near field, the window response characteristics of DAS causes a non-zero systematic shift in the sound source localization results. Especially in the very near field edge region shown in Figure 15e,h (where the sound source distance is comparable to the array aperture), directly estimating the spatial spectrum based on the original DAS output signal will introduce a systematic error with a mean of approximately 5.5 m and a normal distribution.
2. Observing Figure 15b,e,h,k, when using the reconstructed signal for spatial spectrum estimation, the error distribution changes from the original biased normal distribution to an unbiased, peaked distribution. This result demonstrates that Wiener deconvolution effectively eliminates systematic errors introduced by the deviation of DAS window response characteristics, bringing the error distribution closer to zero mean and exhibiting typical random error characteristics.
3. In Figure 15n,o, the vertical distance from the sound source to the array exceeds twice the array aperture. At this point, Wiener filtering deconvolution still has a systematic error calibration effect (Figure 15n), but its effect is weakened compared to when the sound source distance is closer.

## 5. Discussion

### 5.1. On Deriving the PSF Model

Within development of the ϕ-OTDR interrogation system, the EDFA response should be recorded under room temperature (e.g., 25 °C) with the EDFA fully preheated, using all feasible pulse durations and repetition frequencies, in order to achieve optimal deconvolution/resolution reconstruction performance. When the selected parameters exceed the design boundaries, or when environmental conditions biased too much, falling back to the rectangular pulse assumption with a larger NSR provides a suboptimal yet stable deconvolution process, which remains a viable alternative.

### 5.2. On Choosing the NSR Parameter

The selection of the noise-to-signal ratio (NSR) parameter in Wiener filtering is critical for achieving an optimal balance between noise suppression and deconvolution effectiveness. Underestimating the NSR leads to excessive noise amplification, whereas a conservative estimate ensures stable and reliable signal recovery. This inspires a key practical guideline: in engineering applications, a prudent, slightly overestimated NSR is preferable to mitigate the risk of noise-driven artifacts.

The intensity of Rayleigh backscattered light decreases progressively with increasing distance from the fiber input, leading to a corresponding reduction in the average SNR of the acoustic signal. This attenuation also follows a predictable pattern, which can be leveraged to design a distance-aware adaptive strategy for selecting the NSR parameter. This design prevents the NSR from being underestimated due to light intensity attenuation at long distances, thus enhancing numerical stability in long-distance applications. However, this approach may cause the spatial response to vary with space, which some applications may not want.

The noise floor of a DAS system is widely regarded as one of the most critical performance metrics, as it essentially determines the minimum intensity of acoustic events that the system can detect. This metric is influenced by multiple factors, among which laser linewidth and phase noise constitute significant sources of noise in DAS [17,18]. With the Wiener deconvolution approach, a lower noise floor enables the selection of a more aggressive NSR parameter, which in turn enhances the achievable spatial resolution of the system. This establishes a link between the spatial resolution and the performance of the laser source. Exploring this emerging relationship, particularly how laser characteristics ultimately govern the end-to-end resolution after reconstruction, represents a potential direction for future research.

### 5.3. Conclusions of the Experiments

Wiener deconvolution effectively enhances the spatial resolution of DAS by compensating for the spatial averaging inherent in its extended sensing windows. By restoring high spatial frequency components that are otherwise reverted and attenuated, this method improves the system’s ability to distinguish closely spaced acoustic events along the fiber. This constitutes a principled approach to more fully exploiting the spatial information contained in DAS array signals.

In the context of near-field SRP-PHAT-based spatial spectrum estimation, applying Wiener deconvolution prior to processing systematically reduces localization errors and increases positioning confidence. The improvement stems from the correction of phase biases introduced by the spatially blurred system response, which otherwise distort TDoA estimates. Monte-Carlo analyses confirm that this enhancement is consistent and non-accidental, underscoring the value of deconvolution as a preprocessing step for high-fidelity acoustic source localization using linear DAS arrays.

### 5.4. Implication of the Study

We believe this research has the following implications and engineering application value:

1. This study provides an analysis of the causes underlying the spatial window response in rectangular-pulse, heterodyne ϕ-OTDR, explaining why the spatial resolution of DAS cannot be characterized by a single parameter, and offers a theoretical model and an approximate measurement method for the DAS spatial window response.
2. This study provides a single-parameter method to restore DAS’s spatial windowed response as closely as possible toward an ideal point response, and quantitatively evaluates its effects on spatial resolution and acoustic phase recovery in both one-dimensional and two-dimensional sound field measurements.
3. This study provides a sensing characteristic analysis of using linear DAS arrays for SRP-PHAT spatial spectrum estimation. It clarifies that phase deviations caused by the window response introduce systematic errors in the spatial spectrum, and offers an analysis of using Wiener deconvolution to correct such errors.

### 5.5. Limitations of the Study

We believe this study has the following limitations that require further study.

1. The PSF model derived in this paper is based on a common optical configuration using rectangular pulses and heterodyne detection ϕ-OTDR. DAS can also be implemented through multiple optical principles, such as dual pulse [19,20], homodyne detection [21] and optical frequency domain reflectometry (OFDR) systems [22]. These principles utilize different pulse shapes and differential operators. Especially with pulse compression techniques, the shape of the main lobe after template-matched filtering may deviate significantly from the raw EDFA dynamics; the approach presented in this paper may be useful in suppressing spatial response degradation in other DAS implementations, but the PSF model in this paper is not universal.
2. While the deconvolution method used in this study can address spatial blurring and phase reversal issues, nulls still exist in the amplitude–frequency characteristics of the spatial window function; these nulls occur at specific axial spatial wavenumbers determined by the inverse of the window length. For a plane acoustic wave, the axial wavenumber depends jointly on acoustic frequency and incidence angle (when η equals exactly to π,2π,3π… in Equation (47)). As a result, acoustic waves from specific incident directions and frequencies remain undetectable and cannot be recovered by deconvolution or any post-processing technique. These unrecoverable bands therefore represent a fundamental structural limitation imposed by finite spatial averaging rather than an algorithmic deficiency. Addressing this issue may necessitate the use of multi-gauge measurements coupled with data fusion techniques [4].
3. The spatial spectrum estimation algorithm employed in this study is SRP-PHAT, which is based on the GCC principle for TDoA estimation in the frequency domain. Other different methods, such as MUSIC, were not evaluated in terms of the impact of the window response, and further research is needed in this direction.

### 5.6. Perspective and Final Thoughts

It is well established that spatial resolution and SNR in DAS systems are mutually constrained, requiring careful trade-offs in engineering applications. However, a frequently overlooked challenge arises from the secondary effects induced by the spatial window response. The inherent sensing characteristics of DAS make it particularly suitable for adopting deconvolution techniques from imaging problems to address spatial blurring issues in acoustic sensing. Such interdisciplinary approaches may also inspire similar methodologies in other sensing domains.

Notably, the Wiener filtering algorithm features a regular and structured dataflow, primarily consisting of fast Fourier transforms, complex multiplications and divisions, rendering it highly hardware-efficient. If this deconvolution method could be implemented in hardware and integrated into the demodulation circuitry of the DAS interrogation unit, like deblurring algorithms being embedded within image signal processor pipelines, it would enable simple, low-cost DAS systems to output measurements with enhanced spatial resolution and clearer spatial structures in real time. This represents a promising direction for advancing the practical engineering capabilities of DAS technology.

## Figures and Tables

**Table 1:** Correlation and FWHM analysis results between the point-spread-function model response and the measured response.

Metrics	$L_{p}=$ 10 m $L_{g}=$ 11 m	$L_{p}=$ 10 m $L_{g}=$ 20 m	$L_{p}=$ 20 m $L_{g}=$ 21 m
Pearson correlation coefficient	0.9457	0.9637	0.9578
FWHM of Model	11.50	19.27	22.00
FWHM of Measurement	10.18	19.97	≈23.20

**Table 2:** Hardware resource utilization estimation for Wiener deconvolution operator.

Stage	Components	Resources
FFT ^1^	Butterfly operation	396 kbits BRAM, 15 DSP48
	Control logic	~200 LUTs and FFs
Wiener filtering	2 FIR cores	18 DSP slices
	Complex multiplication	24 DSP48 for parallelization
	Filter coefficients	2.4 kbits BRAM
	Control logic	~200 LUTs and FFs
IFFT		Same as FFT ^2^

BRAM: block random access memory; DSP: digital signal processing; LUT: look-up table; FF: flip-flop. ^1^ Estimated in Vivado design suite, version 2022. ^2^ FFT and IFFT share the same butterfly structure and differ only in the sign of the twiddle factors.

**Table 3:** Correlation and FWHM analysis results between the point spread function model response and the measured response.

Selected NSR	Minimum Distinguishable Event Distance	Ratio Relative to Unprocessed Signal
Unprocessed	20.0 m	100.0%
0.5	18.3 m	91.5%
0.3	17.2 m	86.0%
0.1	14.3 m	71.5%
0.05	12.4 m	62.0%
0.03	8.7 m	43.5%
0.01	7.4 m	37.0%

**Table 4:** Comparison of Pearson correlation coefficients between the original DAS output and the Wiener deconvolution output.

Signal Type and Dimension	300 Hz (Spatial Blur)	400 Hz (Phase-Reversal Crossover)	600 Hz (Coherent Cancellation)
**DAS measurement**			
Avg. corr. in time dimension	0.8493	0.6978	0.4927
Avg. corr. in space dimension	0.7996	−0.0611	−0.0886
**Deconvolution output**			
Avg. corr. in time dimension	0.9348	0.9070	0.8325
Avg. corr. in space dimension	0.8178	0.8255	0.5191

**Table 5:** Parameters for spatial spectrum estimation numerical simulation.

Parameter Type	Parameter	Value
Cable	Radial compression ratio	4:1
Optical receiver	ADC sampling rate	250 MSa/s
	SSI on fiber	0.4 m
	SSI on cable	0.1 m
Modulation	Pulse duration	96.6 ns
	Pulse width on fiber	10 m
	Pulse width on cable	2.5 m
Phase demodulation	Gauge length on fiber	20 m
	Gauge length on cable	5 m
	Acoustic recording length	400 ms
	Average acoustic SNR	10 dB
Postprocessing	Wiener filter NSR	0.1

## Data Availability

The data that support the findings of this study are available from the corresponding author upon reasonable request.

## Abbreviations

The following abbreviations are used in this manuscript:

ϕ-OTDR	Phase-sensitive optical time domain reflectometry
ADC	Analog-to-digital converter
AOM	Acousto-optic modulator
DAS	Distributed acoustic sensing
DC	Direct current
DS	Delay-and-sum (beamforming)
DSO	Digital storage oscilloscope
EDFA	Erbium-doped fiber amplifier
FIR	Finite impulse response
FWHM	Full-width-at-half-maximum
GCC	Generalized cross-correlation
LTI	Linear time-invariant (system)
MUSIC	Multiple Signal Classification
NSR	Noise-to-signal ratio
OFDR	Optical frequency domain reflectometry
PSF	Point spread function
RBS	Rayleigh back-scattering
RF	Radio frequency
SNR	Signal-to-noise ratio
SRP	Steered response power
SRP-PHAT	Steered response power—phase transform
SSI	Spatial sampling interval
TDoA	Time difference of arrival
UUT	Unit under test

## Appendix A. PSF Derivation Considering Intra-Pulse Illumination Non-Uniformity

Let $h_{\Sigma }\left(z^{\prime }\right)$ denote the convolution of the pulse and the differential window effect:

(A1) $$h_{\Sigma }\left(z^{\prime }\right)=h_{E}\left(z^{\prime }\right)*h_{g}\left(z^{\prime }\right)=\int_{-\infty }^{\infty }h_{E}\left(\tau \right)\;h_{g}\left(z-\tau \right)\;d\tau .$$

Substituting z−τ into Equation (4) gives

(A2) $$h_{g}\left(z-\tau \right)=\frac{1}{L_{g}}\left[\delta \;\left(z-\tau +\frac{L_{g}}{2}\right)-\delta \;\left(z-\tau -\frac{L_{g}}{2}\right)\right],$$

hence

(A3) $$\begin{array}{cc} h_{\Sigma }\left(z^{\prime }\right) & =\frac{1}{L_{g}}\int_{-\infty }^{\infty }h_{E}\left(\tau \right)\left[\delta \;\left(z-\tau +\frac{L_{g}}{2}\right)-\delta \;\left(z-\tau -\frac{L_{g}}{2}\right)\right]d\tau \end{array}$$

(A4) $$\begin{array}{cc} \; & =\frac{1}{L_{g}}\left[h_{E}\left(z+\frac{L_{g}}{2}\right)-h_{E}\left(z-\frac{L_{g}}{2}\right)\right]. \end{array}$$

Since the non-zero region of $h_{E}\left(z\right)$ is $\left[-\frac{L_{p}}{2},\frac{L_{p}}{2}\right]$, we have $h_{E}\;\left(z+\frac{L_{g}}{2}\right)\neq 0$ if and only if

(A5) $$-\frac{L_{p}}{2}\leq z+\frac{L_{g}}{2}\leq \frac{L_{p}}{2},$$

and $h_{E}\;\left(z-\frac{L_{g}}{2}\right)\neq 0$ if and only if

(A6) $$-\frac{L_{p}}{2}\leq z-\frac{L_{g}}{2}\leq \frac{L_{p}}{2}.$$

Consequently, the non-zero region of $\left(h_{E}*h_{g}\right)\left(z\right)$ is

(A7) $$z\in \left[-\frac{L_{p}+L_{g}}{2},\frac{L_{p}+L_{g}}{2}\right].$$

The integral in Equation (A4) can be written as

(A8) $$h_{\Sigma }\left(z^{\prime }\right)=\left\{\begin{array}{cc} \frac{1}{L_{g}}\left[h_{E}\;\left(z^{\prime }+\frac{L_{g}}{2}\right)-h_{E}\;\left(z^{\prime }-\frac{L_{g}}{2}\right)\right] & \mathrm{for}\;z\in \left[-\frac{L_{p}+L_{g}}{2},\frac{L_{p}+L_{g}}{2}\right],\\ 0 & \mathrm{otherwise}. \end{array}\right.$$

According to the system response from strain to phase (the integral relationship), letting the system PSF be $h_{\mathrm{EP}}$, we obtain

(A9) $$\begin{array}{cc} h_{\mathrm{EP}}\left(z^{\prime }\right) & =\int_{-\infty }^{z^{\prime }}\left(h_{E}*h_{g}\right)\left(z\right)\;dz \end{array}$$

(A10) $$\begin{array}{cc} \; & =\frac{1}{L_{g}}\int_{-\infty }^{z^{\prime }}\left[h_{E}\left(z+\frac{L_{g}}{2}\right)-h_{E}\left(z-\frac{L_{g}}{2}\right)\right]dz. \end{array}$$

Introducing the substitutions

(A11) $$u=z+\frac{L_{g}}{2},\;v=z-\frac{L_{g}}{2},$$

we have

(A12) $$\int_{-\infty }^{z^{\prime }}h_{E}\;\left(z+\frac{L_{g}}{2}\right)dz=\int_{-\infty }^{z^{\prime }+\frac{L_{g}}{2}}h_{E}\left(u\right)\;du,$$

(A13) $$\int_{-\infty }^{z^{\prime }}h_{E}\;\left(z-\frac{L_{g}}{2}\right)dz=\int_{-\infty }^{z^{\prime }-\frac{L_{g}}{2}}h_{E}\left(v\right)\;dv.$$

Thus the integral becomes

(A14) $$\int_{-\infty }^{z^{\prime }}\left(h_{E}*h_{g}\right)\left(z\right)\;dz=\frac{1}{L_{g}}\left[\int_{-\infty }^{z^{\prime }+\frac{L_{g}}{2}}h_{E}\left(u\right)\;du-\int_{-\infty }^{z^{\prime }-\frac{L_{g}}{2}}h_{E}\left(v\right)\;dv\right].$$

Let $H_{E}\left(z\right)$ be the cumulative distribution function of $h_{E}\left(z\right)$:

(A15) $$H_{E}\left(z\right)=\int_{-\infty }^{z}h_{E}\left(\tau \right)\;d\tau ,$$

with

(A16) $$H_{E}\;\left(-\frac{L_{p}}{2}\right)=0,\;H_{E}\;\left(\frac{L_{p}}{2}\right)=1.$$

The system point spread function can then be expressed as

(A17) $$\begin{array}{cc} h_{\mathrm{EP}}\left(z\right) & =\int_{-\infty }^{z}\left(h_{E}*h_{g}\right)\left(\zeta \right)\;d\zeta \end{array}$$

(A18) $$\begin{array}{cc} \; & =\frac{1}{L_{g}}\left[H_{E}\;\left(z^{\prime }+\frac{L_{g}}{2}\right)-H_{E}\;\left(z^{\prime }-\frac{L_{g}}{2}\right)\right]. \end{array}$$

Writing Equation (A17) in piecewise form yields

(A19) $$h_{\mathrm{EP}}\left(z\right)=\left\{\begin{array}{cc} 0 & \mathrm{if}\;z+\frac{L_{g}}{2}<-\frac{L_{p}}{2},\\ \frac{H_{E}\;\left(z+\frac{L_{g}}{2}\right)-H_{E}\;\left(z-\frac{L_{g}}{2}\right)}{L_{g}} & \mathrm{if}\;-\frac{L_{p}}{2}\leq z+\frac{L_{g}}{2}\leq \frac{L_{p}}{2},\\ 0 & \mathrm{if}\;z-\frac{L_{g}}{2}>\frac{L_{p}}{2}. \end{array}\right.$$

## Appendix B. SRP-PHAT Spatial Spectrum Estimation Algorithm

SRP-PHAT is a classic, simple, and effective spatial spectrum estimation method. Typical microphone array signal processing uses Fresnel or Fraunhofer models to simplify near-field and far-field distance calculations. However, DAS has a much larger array aperture than an “acoustic camera”, necessitating the use of an accurate distance model, which may differ from the calculation process in other literature. To facilitate reader reproduction, a brief introduction to the SRP-PHAT algorithm is provided here.

To estimate the signal power at a given spatial location, one can take the time-averaged power of the output of a DS beamformer:

(A20) $$\begin{array}{cc} P_{\mathrm{DS}}\left(r\right) & =E\{{\left|y(r,t)\right|}^{2}\} \end{array}$$

(A21) $$\begin{array}{cc} \; & =E\{y\left(r,t\right)\;y^{*}\left(r,t\right)\}. \end{array}$$

Substituting the DS beamformer

(A22) $$y\left(r,t\right)=\sum_{m=1}^{M}x_{m}\left(t-\tau_{m}\left(r\right)\right)$$

into Equation (A20) and expanding the product gives

(A23) $${\left|y(r,t)\right|}^{2}=\sum_{m}\sum_{n}x_{m}\left(t-\tau_{m}\right)\;x_{n}^{*}\left(t-\tau_{n}\right).$$

Taking the expectation of the above expression yields

(A24) $$P_{\mathrm{DS}}\left(r\right)=\sum_{m,n}E\left\{x_{m}\left(t-\tau_{m}\right)\;x_{n}^{*}\left(t-\tau_{n}\right)\right\}=\sum_{m,n}R_{mn}\left(\tau_{m}\left(r\right)-\tau_{n}\left(r\right)\right),$$

where $R_{mn}$ is the cross-correlation function of channels *m* and *n*.

This derivation shows that the output power of the DS beamformer implicitly accumulates the cross-correlation functions at the same relative delay. Statistically, the algorithm accumulates the coherence between channel pairs. The key idea of the SRP-PHAT algorithm is to exploit this implicit cross-correlation structure to directly compute the spatial response of the array.

The time-domain signals acquired by the *m*-th and *n*-th receiving channels of the array can be written as

(A25) $$x_{m}\left(t\right),\;x_{n}\left(t\right),$$

and their frequency-domain representations as

(A26) $$X_{m}\left(\omega \right),\;X_{n}\left(\omega \right).$$

GCC obtains a correlation measure by weighting the signals in the frequency domain before inverse transformation, thereby emphasising relevant signal components. The generalised cross-correlation function is defined as

(A27) $$R_{mn}\left(\tau \right)=\frac{1}{2\pi }\int_{-\infty }^{\infty }\Phi_{mn}\left(\omega \right)\;X_{m}\left(\omega \right)\;X_{n}^{*}\left(\omega \right)\;e^{j\omega \tau }\;d\omega ,$$

where $\Phi_{mn}\left(\omega \right)$ is a frequency-domain weighting function whose choice determines the performance characteristics of the GCC. GCC performs weighting in the frequency domain, which can effectively enhance the target signal and suppress interference, making it more suitable for time-delay estimation in noisy signals. Phase transform (PHAT) is a GCC weighting function that aims to improve the robustness of delay estimation by suppressing amplitude differences and retaining only phase information. The PHAT weighting function is defined as

(A28) $$\Phi_{mn}^{\mathrm{PHAT}}\left(\omega \right)=\frac{1}{\left|X_{m}\left(\omega \right)\;X_{n}^{*}\left(\omega \right)\right|}.$$

Substituting this into the GCC expression (Equation (A27)) gives

(A29) $$R_{mn}^{\mathrm{PHAT}}\left(\tau \right)=\frac{1}{2\pi }\int_{-\infty }^{\infty }\frac{X_{m}\left(\omega \right)\;X_{n}^{*}\left(\omega \right)}{\left|X_{m}\left(\omega \right)\;X_{n}^{*}\left(\omega \right)\right|}\;e^{j\omega \tau }\;d\omega .$$

This weighting shifts the focus of the beamformer from the amplitude of the summed signal to whether signals from different spatial locations satisfy the same propagation geometry, leading to better performance in complex acoustic environments such as those with reverberation or colored noise.

SRP is a peak-search-based source localization method. The SRP algorithm traverses a predefined grid of candidate source positions and constructs a function that depends on spatial location. For each candidate point in the grid, the algorithm computes the sum of the cross-correlation contributions of all microphone pair signals under the assumption that the source is at that point, ultimately forming a spatial spectrum that reflects the probability distribution of the source.

For each scanning grid point $r_{\mathrm{scan}}$, the theoretical propagation time difference between that point and the *m*-th and *n*-th channels of the array is

(A30) $$\tau_{mn}\left(r\right)=\frac{∥r-r_{m}∥-∥r-r_{n}∥}{c},$$

where *c* is the speed of sound in the propagation medium, and $r_{m}$, $r_{n}$ are the channel positions.

Evaluating the cross-correlation function at the theoretical arrival-time difference and summing over all array channel pairs yields the power–response function at position r, i.e., the SRP value:

(A31) $$P_{\mathrm{SRP}}\left(r\right)=\sum_{\begin{array}{c} \mathrm{all}\;\mathrm{channel}\;\mathrm{pairs}\\ m,n\;(m<n) \end{array}}R_{mn}\left(\tau_{mn}\left(r\right)\right).$$

When the PHAT-weighted GCC is used, the above expression constitutes the SRP-PHAT algorithm, and the spatial spectrum of the acoustic field can be written as

(A32) $$P_{\mathrm{SRP}}^{\mathrm{PHAT}}\left(r\right)=\sum_{\begin{array}{c} \mathrm{all}\;\mathrm{channel}\;\mathrm{pairs}\\ m,n\;(m<n) \end{array}}R_{mn}^{\mathrm{PHAT}}\left(\tau_{mn}\left(r\right)\right).$$

By scanning $P_{\mathrm{SRP}}^{\mathrm{PHAT}}$ over a predefined spatial search region, a spatial spectrum distribution is obtained. The peak locations correspond to the estimated source positions, and the number of peaks reflects the number of potential sources.
